# Initial loss to follow up of tuberculosis patients in South Africa: perspectives of program managers

**DOI:** 10.1186/s12889-020-08739-w

**Published:** 2020-05-06

**Authors:** Judith R. M. Mwansa-Kambafwile, Sara Jewett, Charles Chasela, Nazir Ismail, Colin Menezes

**Affiliations:** 1 Department of Epidemiology and Biostatistics, School of Public Health, Faculty of Health Sciences, University of the Witwatersrand, Johannesburg, South Africa; 2 Centre for Tuberculosis, National Institute of Communicable Diseases, Johannesburg, South Africa; 3 Fellow of the Consortium for Advanced Research Training in Africa (CARTA), Johannesburg, South Africa; 4 Division of Health and Society, School of Public Health, Faculty of Health Sciences, University of the Witwatersrand, Johannesburg, South Africa; 5 Right to Care, Johannesburg, South Africa; 6 Department of Microbiology, University of Pretoria, Pretoria, South Africa; 7 Department of Internal Medicine, School of Clinical Medicine, Faculty of Health Sciences, University of Witwatersrand, Johannesburg, South Africa; 8 Department of Internal Medicine, Chris Hani Baragwanath Academic Hospital, Johannesburg, South Africa

**Keywords:** TB treatment, Initial loss to follow up, Program managers, South Africa

## Abstract

**Background:**

Tuberculosis (TB) remains a serious public health problem in South Africa. Initial loss to follow up (LTFU) rates among TB patients are high, varying between 14.9 and 22.5%. From the perspective of patients, documented reasons for this include poor communication between patient and staff after testing, not being aware that results are ready and other competing priorities such as preference to go to work as opposed to seeking healthcare. Ward-based Outreach Teams (WBOTs) routinely conduct home visits to ensure adherence to medication for various conditions including TB. We explored reasons for TB initial loss to follow up from the perspectives of TB program managers and WBOT program managers, with a focus on the WBOT’s (potential) role in reducing initial LTFU, in particular.

**Methods:**

Key informant interviews with five WBOT program managers and four TB program managers were conducted. The interviews were audio-recorded, then transcribed and exported to NVivo 11 software for coding. A hybrid analytic approach consisting of both inductive and deductive coding was used to identify themes.

**Results:**

The age of the nine managers ranged between 28 and 52 years old, of which two were male. They had been in their current position for between 2 to 12 years. Prior to treatment initiation, WBOTs screen household members for TB and refer them for TB testing if need be, but integration of the two programs is emphasized only after TB treatment has been initiated. Counseling of patients testing for TB is not guaranteed due to frequent staff rotations and staff shortages. Participants reported that possible dissatisfaction with services as well as stigma associated with the TB diagnosis could explain loss to follow up prior to treatment initiation.

**Conclusion:**

Program managers view health system related factors such as staff rotations, poor communication with patients and lack of counseling as contributing to the problem of initial LTFU among TB patients. The integration of the WBOT and TB programs is limited to referring suspected cases for testing and patients already on treatment.

## Background

Although mortality due to tuberculosis (TB) in South Africa has been declining, the disease still tops the list of the “ten leading underlying natural causes of death, 2014–2016” [1]. The incidence of this deadly yet preventable disease in South Africa is currently 567/100000 population in the general population and 340/100000 among individuals living with HIV [2]. TB patients already on treatment have been a focus of TB control programs in many countries including South Africa. The emphasis has mostly been on ensuring that patients take their treatment. This has been evidenced from the implementation of directly observed therapy short-course (DOTS).

In South Africa, a patient with presumptive TB in whom a productive cough is among the presenting symptoms is asked to provide a sputum sample for laboratory testing using Xpert MTB/Rif (Xpert), culture or smear-microscopy testing based on the screening algorithm. Although laboratory turnaround time for Xpert is 2 h, the patient is asked to come back after 2 days to cater for transportation time and delivery of result to the facility. When the patient returns and if Xpert TB test result is positive, treatment is supposed to be initiated on the same day or within 5 days [3]. The patient is also asked to submit another sputum sample for smear microscopy as a baseline for monitoring treatment progress. Patients who test positive for TB but never get initiated on treatment are known as initial loss to follow up (LTFU) patients [3].

Initial LTFU rates in South Africa range between 14.9 and 18.0% [4, 5]. Although this upper limit rate corroborates the rate found in African studies in an earlier systematic review [6], a higher rate of 22.5% was found in another study conducted in inner city Johannesburg, South Africa [7]. This city is home to economic immigrants from other parts of the country as well as from other countries [8]. A study conducted in the Western Cape province, a similar economic hub in South Africa, highlighted mobility and temporary migration as the most common reasons for missed appointments among patients on chronic medication because generally people travel to their homes of origin during the festive season or travel on other planned holidays [9].

There are various reasons for non-initiation of treatment for patients who test positive for TB. Breakdowns in communication between patients and providers are one reason why patients with varying conditions do not show up for appointments at healthcare facilities. A study conducted in different clinical departments at a regional hospital in South Africa found that at least 16% were unaware of their appointment date while 11% were unsure of when their actual appointment date was [10]. The lack of proper communication between patient and healthcare provider with regards to next steps was also a cause for initial LTFU in India’s TB program [11], showing that the problem is not unique to South Africa. Findings from two other studies conducted in India and Pakistan revealed that patients were not aware that their results were ready at the facilities [12, 13] suggesting a breakdown in communication between the patients and the providers with regards to next steps at the time of testing. Poor counseling and general poor healthcare worker attitudes have been shown to contribute to patients being lost to follow up [14]. Other reasons for not starting treatment are related to quality of the healthcare services rendered to patients either in form of structural barriers or lack of proper recording and reporting [15–17].

Patient related factors are also a cause for initial loss to follow up. “Being busy with other jobs” was found to be the main reason for non-initiation of treatment in a study conducted in India [18]. Lack of motivation for a second visit and having other competing priorities are also reasons for TB patients not getting initiated on treatment [12, 19]. Other reasons reported are re-treatment, changing residence, feeling ashamed and alcohol consumption [14, 20, 21]. Poverty, lack of education, not having someone to go with to the clinic, consulting traditional healers, social stigma and religious beliefs also contribute to initial LTFU [15, 22, 23].

In countries like Brazil (with a similar GDP as South Africa), the use of community healthcare workers to take healthcare services to the community has been shown to be beneficial by improving access to services [24] and by reducing infant mortality rate [25]. Within the structure of South Africa’s re-engineered primary healthcare (PHC) model are ward-based outreach teams (WBOTs). The WBOTs consist of a Team Leader (often a Professional Nurse but can be an Enrolled Nurse) and 6 Community Healthcare Workers (CHWs) including a Health Promoter (HP) and an Environmental Health Officer (EHP) [26]. The WBOTS work within specific geographical areas of PHC facility total catchment areas. Their scope of work is centred on health promotion and prevention of disease. In the TB program, screening, tracing and treatment adherence support is more of WBOTs’ role than treatment initiation. This is part of their work and therefore no incentives are provided for doing this work [27].

The strategy of the South African TB control program is guided by strategies developed by the Department of Health for tackling HIV, STI and TB and is collectively known as HAST. HAST programs at the various levels of governance are managed by HAST managers. Historically, staff supervising TB programs were known as TB managers. Thus, HAST managers who were tasked to supervise the HIV and STI programs became collectively known as TB managers.

With regards to TB services, the WBOTs’ work is to identify, support and follow-up TB patients and their contacts [26]. We explored reasons for TB initial loss to follow up from the perspectives of TB program managers and WBOT program managers, with a focus on the WBOT’s (potential) role in reducing initial LTFU, in particular.

## Methods

This exploratory qualitative study was part of a bigger study looking at interventions to reduce initial loss to follow up among TB patients. The study is entitled “Initial Loss to Follow Up Among Tuberculosis Patients: The Role of Ward-Based Outreach Teams and Short Message Service (SMS) Technology” [27]. In depth interviews (IDIs) with TB/WBOT program managers were conducted in the City of Johannesburg district in South Africa between August 2018 and February 2019. This city is inhabited predominantly by migrants (both national and regional) [8]. This is a risk factor for loss to follow up of health services. Knowing more about loss to follow up in this region would assist the TB program nationally because South Africa as a whole serves as an economic hub, not only for the Southern African region, but for Africa at large. We defined WBOT Managers as regional coordinators or outreach team leaders for the WBOT program; and TB Managers were the HAST Managers for respective regional areas. The research and its objectives were shared with the respective directorates of the district who then provided names and contact details of 20 managers we could invite to participate in the research. A maximum of three telephonic attempts and one email over a period of 5 days were made to arrange an appointment before declaring a potential participant “unable to reach.” The participants were conveniently selected depending on their availability. Of the twenty managers on the list provided, seven could not be reached and one manager who was reached by email said he had relocated out of the country. Nine of the twelve managers approached for participation accepted the invitation telephonically. The three who did not participate gave the following reasons for their unavailability: annual leave, illness and other commitments. All interviews were conducted in English by the first author in the privacy of the participants’ respective offices after written informed consent was obtained.

With the participants’ verbal permission, the IDIs were audio-recorded, lasting on average about 35 min each. The interviewer also took notes. To ensure standardization of topics discussed, an interview guide was developed and used. As prompts to elicit discussions, the guide included the following topic areas: TB communication, WBOT functions and reasons for loss to follow up. Prior to data collection, this instrument was piloted with two managers working in programs other than the TB or WBOT programs and then refined before a final version was agreed upon. These data are not presented. The purpose of the pilot was to ensure that the questions were clear with no ambiguity in them. We wanted to know how best to phrase the questions to ensure clarity as well as the average time an interview would take before scheduling interview appointments.

The audio recordings were transcribed verbatim and the transcripts were systematically coded by the first author using NVivo 11 software [28]. A codebook framework of the topic areas was created by the first author. The second author checked the reliability of the coding framework by recoding four of the transcripts using the framework. After this, a hybrid approach [29] was applied to code each transcript, first inductively and then according to deductive codes derived from factors identified in the literature. These were the grouped into themes aligned with the different concepts/topic areas.

## Results

Five WBOT managers and four TB managers (a.k.a. HAST managers) participated in the study. Participants were between 28 and 52 years of age and seven of the nine were female. Work experience in current position ranged from 2 to 12 years (Table 1).

**Table 1:** Characteristics of IDI participants

Participant	Gender	Program Area	Duration in current position (years)
IDI 1	Female	HAST Program Manager	12
IDI 2	Male	HAST Program Manager	3
IDI 3	Female	WBOT Program Manager	10
IDI 4	Female	HAST Program Manager	7
IDI 5	Female	WBOT Program Manager	3
IDI 6	Female	WBOT Program Manager	2
IDI 7	Female	WBOT Program Manager	11
IDI 8	Male	WBOT Program Manager	8
IDI 9	Female	HAST Program Manager	6

Emerging themes were grouped under the following areas: TB program knowledge, WBOT functions and reasons for initial LTFU.

### Knowledge of the TB program

#### WBOTs don’t know how TB program works

The managers in the TB program knew how the program worked and how they work with the WBOTs. They were able to describe, step by step the procedure of treatment initiation. A HAST Program Manager (IDI 1) explained that *“We use GXP for testing. We ask patients to come back after 2 days for results”.* In contrast, WBOT program managers were less clear. They did not know exactly how the TB program worked in terms of the procedure that is taken when a patient is found to be positive and needs to be put on treatment. *“I…I really don’t know how they work in the TB program,”* explained a WBOT Program Manager who had been in her post for 10 years (IDI 3).

### WBOT functions

#### Nature of work

The WBOTs offer a wide range of services when they do the home visits. In terms of TB service delivery, the WBOTs screen household members for TB as they conduct routine home visits for chronic conditions likes diabetes and hypertension. Any household members with TB symptoms (cough, night sweats, fever, weight loss, etc.) is referred to the nearest facility for TB testing. At the facility, the TB nurse collects sputum from patient and sends to the laboratory for TB testing. A male WBOT Program Manager (IDI 8) had the following to say:

“What they do when they go to the household is uuuhhmm…they do screening for TB and they refer patients for TB testing. That’s all they do”*.*

Participants also generally reported that the work of the WBOTs with regards to the TB program starts once a patient is initiated on TB treatment. A WBOT Program Manager with more than 10 years’ experience in her program area (IDI 7) explained this:

“Those who have already started treatment but default along the way, those are the ones we focus on and we go out and trace them. But those ones who come to test and are told to come back for results...no those we do not focus on.”

#### Working together

While TB program procedures were unclear to WBOT Program Managers, both groups of managers understood where their roles overlapped.

For patients not yet on TB treatment, everyone knew that WBOTs are involved in screening of household members for TB during their routine home visits, as reported earlier. There was then a gap between referral and TB patients being initiated on treatment, which is the period when initial LTFU occurs.

WBOTs also support the TB program in offering treatment adherence support during their home visits. During the home visits, they also trace treatment defaulters. The managers described how TB and WBOT programs work together when it comes to defaulter tracing of patients already initiated on treatment. A WBOT Program Manager (IDI7) described this process:

“TB people phone and if patient does not come, then they hand over the list of names of these people to the WBOT members who now go into the community to look for them.”

A HAST Program Manager working in her position for 7 years (IDI 4) echoed this description.

“For contacts it’s not easy for them to come and it’s also not easy for us to go to them. But we are working in close contact with the WBOTs.”

There were no descriptions on how the TB program works with WBOTs to confirm referrals or to follow up on patients who had not yet initiated treatment.

### Reasons for initial loss to follow up

#### Poor communication with patients

Participants reported that counseling of TB patients was lacking. A HAST Program Manager with 6 years’ work experience in TB (IDI 9) expressed that counseling should be emphasized in a similar way as done in the HIV program.

“I think it’s probably…patients are not counselled when they are tested…you know like in HIV. So the patients do not know the importance of starting treatment soon. So they just go home. And remember they test and are given a date when to come back and this is where the gap is. They normally don’t come back on their own.”

WBOT program managers also acknowledged that the WBOTs only focus on household visits as part of routine work and deal with patients who had visited the facilities only if they are part of the defaulter list that is given to them by the TB nurse for tracing of such patients. A WBOT Program Manager with 3 years’ experience (IDI 5) reported that

“Mostly we are focusing on the household…so maybe if we also focus on the ones in clinic so that the same message goes everywhere, maybe it can work.”

Apart from counseling of patients on TB, the general communication between nurses and patients with regards to next steps after testing for TB was also unclear. A HAST Program Manager with 12 years’ experience (IDI 1) echoed this and explained that

“………they were not informed. Procedure was not explained to patient”*.*

#### Health system barriers

Both WBOT and TB program managers pointed out that the health system was a significant contributor to the problem of loss to follow up. Patient waiting times at facilities need to be shortened so that this is not used as an excuse for “no-show” by patients. A WBOT district coordinator described how patients *“…give excuses that they are long queues”* as a cause for not starting treatment.

The need for patients to visit clinics to get tested for TB and initiate treatment, if found positive, relates to how nurses’ roles are licensed. Despite WBOTs doing screening during the home visits, as indicated earlier, they are not allowed to conduct testing or initiate treatment. They rely on patients to act upon their referrals to facilities. The WBOTs cannot refer the patients to their team leader, if the latter is an enrolled nurse, for treatment initiation because enrolled nurses cannot prescribe medications in the South African healthcare system.

TB Managers expressed that it is important for all facility staff to get familiar with the TB program so that should the TB nurse not be available patient management is not interrupted. It is also important to keep the staff rotation period in a particular program area for longer than the current 3 months to ensure proper staff training in the respective program area. A HAST Program Manager working in TB for more than 10 years (IDI 1) had this to say

“…And then if someone comes to stand in for the TB sister, they will not attend to the patient thoroughly as they’re supposed to, sometimes not even informing them about importance of ensuring they come back for the results.”

Participants reported that staff generally do not like to work in the TB room and are reluctant to acquaint themselves with systems and processes on TB patient management. As such, when the TB nurse is absent, either the TB room is locked for the day or the substitute staff member does not adequately manage the patients in terms diagnosis and treatment as well as counseling on next steps. Staff rotations which happen quite often also mean that the new person in the TB room has first to acquaint himself/herself before they can be deemed competent in TB patient management. However, it happens in most cases that as soon as they have settled in, it is time for another cycle of staff rotation. Participants suggested less staff rotation in the TB room and TB patient management education to all facility clinical staff so that continuity of optimal TB patient care is guaranteed.

#### Patient responsibilities

Participants reported that patients’ behavior is also a reason for their failure to initiate TB treatment. Patient relocation emerged strongly as a reason. A WBOT Program Manager (IDI 6) reported that *“……..patients are always relocating.”* They move house and probably start seeking healthcare services at a facility close to their new home.

The managers also reported that provision of correct contact details is important as this makes it easier for the healthcare system to find a missing patient. According to the TB managers, patients give wrong phone numbers and wrong addresses. Unfortunately, this is only discovered at the point of defaulter tracing.

Patients “shopping around” for better health services was a reason also given by the managers. A male HAST Program Manager (IDI 2) had this to say:

“They like to confirm and they will come here and test and if they are positive here they will go to another facility to test. Maybe if still not satisfied, they will go to another.”

Sometimes patients get worse after testing, get admitted to hospital and therefore fail to return for the results. At this point, it is important that a relative or friend informs the facility so that the patient is not labelled as LTFU.

## Discussion

The most critical findings from this study relate to how WBOTs may contribute to reductions in initial LTFU in the future. Currently, the role of the WBOTs in the TB program is limited to symptomatic screening and to tracing of patients who default their TB treatment [26]. TB nurses give lists of patients who have missed their appointments by over a week to WBOTs who go and trace these defaulters so that they can continue with their treatment. TB initiation is not among the functions of the WBOTs [26], but it could be added to the enrolled nurse function. Patients not initiated on treatment continue to spread TB in the community and thereby increase the TB burden [30, 31]. In line with task shifting, enrolled nurses in rural clinics of South Africa take on most of the roles of professional nurses [32] with the exception of medicine prescribing. Revision of policy to train and allow enrolled nurses from the WBOT program to prescribe TB drugs would assist in ensuring that patients start TB treatment promptly.

This study reinforced the two-fold reasons for initial LTFU: patient related and health system related. Although the patient-related factors were reported second-hand, they still are worth discussing. For instance, a factor such as relocation is not something the health system can control, but it can create systems to reduce LTFU such as ensuring that quality patient contact information is recorded at the time of TB testing [33]. City of Johannesburg, where the study was conducted, is South Africa’s economic hub and occupied predominantly by economic migrants from both within and outside the country [8]. Although the city occupies an area of 1645km^2^, it has a population of 4.4 million [34]. This dense population could explain the high total headcount numbers and consequent high loss to follow up rates in the healthcare facilities within the city reported in an earlier study [7]. A Malawian study looking at initial LTFU of antiretroviral therapy found that high burden facilities were more likely to have higher initial LTFU rates than low burden facilities and attributed this to inadequate staffing leading to poor patient education and lack of counseling [35]. In light of this and of the association between TB and HIV, it is important that the healthcare facilities be adequately staffed. Task-shifting strategies [32], whereby WBOTs could be involved in treatment initiation (such as explaining to patients on next steps after each consultation) to ensure minimal LTFU among TB patients, should be explored.

Another patient related factor that was reported by participants was that patients are tested at different facilities because they want to confirm the diagnosis. Patients go and test at other facilities to see if the result is the same. This finding is corroborated by a recent study that looked at health seeking pathways of patients with drug resistant tuberculosis [36]. Courtwright and Turner found that some of the causes of TB stigma were: perceived transmission risk from infected people; poverty; and its association with HIV [37].

In terms of health system related factors for patients not initiating treatment, proper and adequate communication with patients is highly essential in ensuring optimal initiation and adherence to treatment. Divija and colleagues reported in their study that poor communication and education to the patient on next steps were reasons for initial LTFU [11]. One of our study participants mentioned that it is important to put emphasis on proper counseling and education of patients with regards to TB as a disease. Therefore, it is expected that with optimal patient communication and counseling, patients would know that they need to inform the facility when they are leaving the facility catchment area. That way they can be referred and documented as such. Our findings suggest that training on counseling may benefit existing TB managers as well as WBOTs.

Good quality data in the TB program ensures optimal control of the disease. The design of TB data collection and reporting systems need to be such that they enable responses at the community level that are specific to gaps identified from the data [38]. Properly documented records which are frequently updated can ensure that all patients have outcomes; and those missing appointments are timeously identified and appropriately followed up by the WBOTs.

Although Professional Nurses are the preferred team leaders, most WBOTs are led by Enrolled Nurses due to shortage of staff at the clinics. Enrolled Nurses are not licensed to prescribe TB treatment. This means traced patients cannot be given treatment unless they go to the clinic.

## Conclusion

Program managers view health system related factors such as staff rotations, poor communication with patients and lack of counseling as contributing to the problem of initial LTFU among TB patients. The integration of the WBOT and TB programs is limited to referring suspected cases for testing and patients already on treatment. We have identified immediate opportunities to improve integration, e.g. engaging WBOTs to follow up with patients who have been tested, but not yet initiated as well as longer-term considerations, such as revisiting licensing rules around enrolled nurses being permitted to initiate TB treatment.

TB initial LTFU can be prevented by addressing health system related factors such as ensuring that patients are counselled, ensuring that competent TB treatment providers are present at all times to attend to patients and implementing policies that reduce stigma and protect TB patients as well as survivors of TB. In addition, there is need for regular meetings between the WBOT and TB programs at various levels of patient care to ensure optimal integration of the two programs.

## Data Availability

The transcripts are not publicly available due to confidentiality agreements with the participants.

## Abbreviations

TB: Tuberculosis
LTFU: Loss to follow up
WBOT: Ward-based outreach team
WHO: World health organization
DOTS: Directly observed therapy short-course
Xpert: Xpert MTB/Rif
PHC: Primary healthcare
CHWs: Community healthcare workers
HP: Health promoter
EHP: Environmental health officer
